# Supplementary material for: Towards unified AI-driven fracture mechanics: the extended deep energy method (XDEM)
Source: Nat Commun. 2026 Aug 15;17:8492. doi: 10.1038/s41467-026-76748-1 (PMC13484471; doi:10.1038/s41467-026-76748-1)
Supplement: Supplementary file 3 [file 41467_2026_76748_MOESM3_ESM.zip › pic/Results/Stress_intensity_factor/Mode1/k1_compare.pdf]

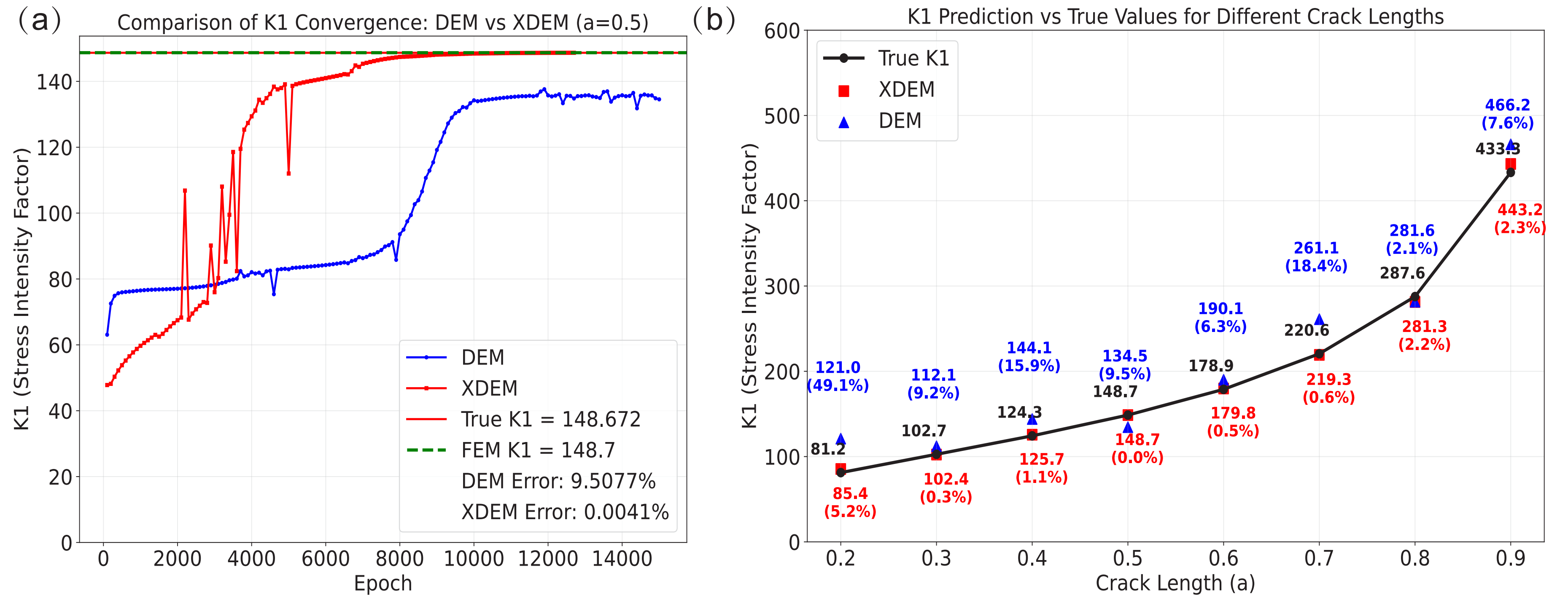

(a)
Comparison of K1 Convergence: DEM vs XDEM (a=0.5)
K1 (Stress Intensity Factor)
Epoch
DEM
XDEM
True K1 = 148.672
FEM K1 = 148.7
DEM Error: 9.5077%
XDEM Error: 0.0041%
(b)
K1 Prediction vs True Values for Different Crack Lengths
K1 (Stress Intensity Factor)
Crack Length (a)
True K1
XDEM
DEM
121.0 (49.1%)
81.2
85.4 (5.2%)
112.1 (9.2%)
102.7
102.4 (0.3%)
144.1 (15.9%)
124.3
125.7 (1.1%)
134.5 (9.5%)
148.7
148.7 (0.0%)
190.1 (6.3%)
178.9
179.8 (0.5%)
261.1 (18.4%)
220.6
219.3 (0.6%)
281.6 (2.1%)
287.6
281.3 (2.2%)
466.2 (7.6%)
433.3
443.2 (2.3%)
